# Association between polygenic risk score of Alzheimer’s disease and plasma phosphorylated tau in individuals from the Alzheimer’s Disease Neuroimaging Initiative

**DOI:** 10.1186/s13195-020-00754-8

**Published:** 2021-01-08

**Authors:** Anna Zettergren, Jodie Lord, Nicholas J. Ashton, Andrea L. Benedet, Thomas K. Karikari, Juan Lantero Rodriguez, the Alzheimer’s Disease Neuroimaging Initiative*, Anniina Snellman, Marc Suárez-Calvet, Petroula Proitsi, Henrik Zetterberg, Kaj Blennow

**Affiliations:** 1 Neuropsychiatric Epidemiology Unit, Department of Psychiatry and Neurochemistry, Institute of Neuroscience and Physiology, the Sahlgrenska Academy, Centre for Ageing and Health (AGECAP) at the University of Gothenburg, Gothenburg, Sweden; 2 Maurice Wohl Clinical Neuroscience Institute, Institute of Psychiatry, Psychology and Neuroscience, King’s College London, London, UK; 3 Department of Psychiatry and Neurochemistry, Institute of Neuroscience & Physiology, the Sahlgrenska Academy at the University of Gothenburg, Mölndal, Sweden; 4 Wallenberg Centre for Molecular and Translational Medicine, University of Gothenburg, Gothenburg, Sweden; 5 Department of Old Age Psychiatry, Maurice Wohl Clinical Neuroscience Institute, King’s College London, London, UK; 6 NIHR Biomedical Research Centre for Mental Health & Biomedical Research Unit for Dementia at South London & Maudsley NHS Foundation, London, UK; 7 Translational Neuroimaging Laboratory, McGill Centre for Studies in Aging, McGill University, Montreal, QC Canada; 8 Turku PET Centre, University of Turku, Turku, Finland; 9 Barcelonaβeta Brain Research Center (BBRC), Pasqual Maragall Foundation, Barcelona, Spain; 10 IMIM (Hospital del Mar Medical Research Institute), Barcelona, Spain; 11 Servei de Neurologia, Hospital del Mar, Barcelona, Spain; 12 Centro de Investigación Biomédica en Red de Fragilidad y Envejecimiento Saludable (CIBERFES), Madrid, Spain; 13 Clinical Neurochemistry Laboratory, Sahlgrenska University Hospital, Mölndal, Sweden; 14 Department of Neurodegenerative Disease, UCL Institute of Neurology, Queen Square, London, UK; 15 UK Dementia Research Institute at UCL, London, UK

**Keywords:** Alzheimer’s disease, Polygenic risk score, Plasma phosphorylated tau 181

## Abstract

**Background:**

Recent studies suggest that plasma phosphorylated tau181 (p-tau181) is a highly specific biomarker for Alzheimer’s disease (AD)-related tau pathology. It has great potential for the diagnostic and prognostic evaluation of AD, since it identifies AD with the same accuracy as tau PET and CSF p-tau181 and predicts the development of AD dementia in cognitively unimpaired (CU) individuals and in those with mild cognitive impairment (MCI). Plasma p-tau181 may also be used as a biomarker in studies exploring disease pathogenesis, such as genetic or environmental risk factors for AD-type tau pathology. The aim of the present study was to investigate the relation between polygenic risk scores (PRSs) for AD and plasma p-tau181.

**Methods:**

Data from the Alzheimer’s Disease Neuroimaging Initiative (ADNI) was used to examine the relation between AD PRSs, constructed based on findings in recent genome-wide association studies, and plasma p-tau181, using linear regression models. Analyses were performed in the total sample (*n* = 818), after stratification on diagnostic status (CU (*n* = 236), MCI (*n* = 434), AD dementia (*n* = 148)), and after stratification on Aβ pathology status (Aβ positives (*n* = 322), Aβ negatives (*n* = 409)).

**Results:**

Associations between plasma p-tau181 and *APOE* PRSs (*p* = 3e^−18^–7e^−15^) and non-*APOE* PRSs (*p* = 3e^−4^–0.03) were seen in the total sample. The *APOE* PRSs were associated with plasma p-tau181 in all diagnostic groups (CU, MCI, and AD dementia), while the non-*APOE* PRSs were associated only in the MCI group. The *APOE* PRSs showed similar results in amyloid-β (Aβ)-positive and negative individuals (*p* = 5e^−5^–1e^−3^), while the non-*APOE* PRSs were associated with plasma p-tau181 in Aβ positives only (*p* = 0.02).

**Conclusions:**

Polygenic risk for AD including *APOE* was found to associate with plasma p-tau181 independent of diagnostic and Aβ pathology status, while polygenic risk for AD beyond *APOE* was associated with plasma p-tau181 only in MCI and Aβ-positive individuals. These results extend the knowledge about the relation between genetic risk for AD and p-tau181, and further support the usefulness of plasma p-tau181 as a biomarker of AD.

## Background

Neuropathological features of Alzheimer’s disease (AD) are defined by aggregation of amyloid-β (Aβ) protein into plaques and hyperphosphorylation of tau protein with the formation of neurofibrillary tangles [1]. Detection of these processes is crucial for diagnosis of the disease and for recruitment of individuals in clinical trials. The availability of cerebrospinal fluid (CSF) and positron emission tomography (PET) biomarkers has been important for clinical and research work in the field [2]. However, methods for measuring these biomarkers have some drawbacks, since they are invasive, time-consuming, and expensive; may have side effects; and have limited availability. It is therefore a great need for more easily accessible biomarkers, such as those measured in plasma. Recent studies have indicated that plasma phosphorylated tau 181 (p-tau181) could be a useful biomarker for AD diagnosis and prognosis. It identifies AD with the same accuracy as tau PET [3–5] and CSF p-tau181 [4] and associates with development of AD dementia in cognitively unimpaired (CU) individuals and in those with mild cognitive impairment (MCI) [4], as well as in carriers of mutations causing familial AD [6]. Furthermore, plasma p-tau181 accurately discriminates AD from non-AD neurodegenerative disorders with post-mortem confirmation [7]. The relation between plasma p-tau181 and genetic risk for sporadic AD is, however, uninvestigated.

Genetic risk for AD is often studied through the use of polygenic risk scores (PRSs), which allow the calculation of genetic risk based on several genetic variants identified through genome wide association studies (GWASs) [8–10]. PRSs for AD have been shown to predict clinical diagnosis [11], pathology-confirmed diagnosis [12], cognitive decline [13], disease progression [14], and imaging biomarkers [13, 15]. Analyses of the association between AD-PRS and CSF biomarkers have shown somewhat mixed results. The most recent study in the Alzheimer’s Disease Neuroimaging Initiative (ADNI) cohort reports that CSF t-tau and p-tau were associated with polygenic scores after correcting for *APOE ε4* status, while the association seen with CSF Aβ42 was driven by *APOE ε4* homozygotes [16].

There is a need to further explore how genetic risk for AD influences specific components of the AD pathophysiology, i.e., brain amyloidosis, tau pathology, and synaptic and neuronal degeneration. Since plasma p-tau181 reflects AD-type tau pathology, it has value as a biomarker to increase understanding on how PRS is linked to tau pathology in AD and thereby increase the knowledge of disease pathogenesis. The aim of the present study was to investigate the relation between AD-PRSs (including and excluding *APOE*) and plasma p-tau181 in ADNI study participants, both in the total sample and after stratifying on diagnostic status and Aβ pathology status.

## Methods

### Data

The data used in this article was obtained from the ADNI database [17]. ADNI was launched in 2003 as a public-private partnership led by Principal Investigator Michael W. Weiner. Briefly, ADNI is a US- and Canada-based longitudinal initiative, following participants through multiple study phases in an attempt to uncover predictive and diagnostic markers of AD [18]. Regional ethical committees of all institutions included in ADNI approved of the study and all subjects have provided informed consent.

### Fluid and imaging biomarkers

Blood samples were collected, processed, stored, and analyzed as described previously [19]. Plasma p-tau181 was measured using the single molecule array (Simoa) technique, using an in-house assay developed in the Clinical Neurochemistry Laboratory, University of Gothenburg, Sweden [3]. This assay uses a combination of two monoclonal antibodies (Tau12 and AT270) and measures N-terminal to mid-domain forms of p-tau181. Further details of the assay can be found in [3, 20]. Brain Aβ load was estimated using [^18^F] florbetapir (Aβ PET) standardized uptake value ratios (SUVR). SUVR volumes were generated using the full cerebellum as reference region for [^18^F] florbetapir and amyloid positivity was defined as SUVR > 1.11 [21]. For this study, we used imaging data with the closest acquisition date to the plasma collection. More specifically, amyloid status was given based on [^18^F] florbetapir scans acquired ± 180 days (mean (SD): 12.8 (22.1)) from the date of baseline blood collection. Alternatively, a positive amyloid PET status was considered if the PET acquisition was performed more than 180 days before baseline blood collection. Finally, a negative amyloid status was attributed if the PET acquisition was performed more than 180 days after baseline blood collection. Cases that did not fulfill these criteria were excluded.

### Genotype data

Data was available across 1674 individuals and three genotyping platforms: (1) the Human610-Quad platform (ADNI1), (2) the HumanOmniExpress (ADNI2), and (3) Omni 2.5M platform (a combination of ADNI1 and ADNI2 typed at high coverage) [22]. Prior to imputation, data from each of these platforms were QC’ed separately using a combination of PLINK 1.9, PLINK 2.0 [23], and R 3.6.1. Briefly, samples were retained if (1) call rates were above 98%, (2) identity-by-decent (IBD) scores were lower than 0.1875 (genetic relatedness less than half-way between second and third degree relatives), (3) clinical and genetic sex information corroborated, and (4) samples were of white European descent. To interrogate ancestry beyond that clinically reported, principal components (PCs) were generated using EIGENSOFT 6.1.4 [24] and superimposed onto chart visualizations generated within R using data from the 1000 genome project [25]. The top 10 PCs were iteratively plotted against each other and visually inspected for each dataset. Upon initial inspection, a sub-cluster of samples were found to cluster independently in PCs explaining the greatest variance within the data. These samples were also found to cluster outside of the European range when superimposed onto 1000 genomes data. Outlier samples were iteratively removed using plot co-ordinates and subsequently re-inspected. Following outlier removal, all data was found to cluster within the European region. Further inspection of sample-only plots indicated a necessity to adjust for the top seven PCs, after which point data was found to fall within a homogenous cluster. Single nucleotide polymorphisms (SNPs), like samples, were retained if call rates were > 98%. Rare SNPs (minor allele frequency (MAF) < 0.01), non-autosomal SNPs, and those deviating from Hardy-Weinberg expectations (*p* value threshold of 0.00001 - controls only) were also removed. Any sample overlaps remaining at the end of QC were removed in accordance with the following criteria: for overlaps between Human610-Quad and Omni 2.5 M, duplicates in Omni 2.5M were removed and Human610-Quad retained; for overlaps between HumanOmniExpress and Omni 2.5M, duplicates in HumanOmniExpress were removed and Omni 2.5M retained, generating a final sample of 1324 individuals.

Imputation was implemented via the Sanger Imputation Service [26], where EAGLE2 was used for pre-phasing and the Haplotype Reference Consortium was utilized as the reference panel [27]. Post-imputation QC consisted of multi-allelic SNP removals and removal of SNPs with an imputation score < 0.4 or with dosage certainty < 0.9. Any retained SNPs were treated as fully observed, and genotypes across the three platforms were merged subsequent to post-imputation QC.

### Polygenic risk scores

Three versions of AD-PRSs were used in this study. To be able to investigate the influence of both “total genetic risk” for AD and genetic risk for AD beyond *APOE*, and to compare our findings with previous studies, all versions were constructed with and without *APOE* included. Two of the versions were generated based on summary statistics from the most recent AD GWAS including pure AD phenotypes [8]. SNPs with MAF ≥ 5% were used for selection by linkage disequilibrium (LD)-clumping. The European ancestry samples from the 1000-genomes project were used as reference panel to remove variants in LD, all variants 250 kb upstream and downstream of the top signal were removed (*R*^2^ < 0.001). For the non-*APOE* PRSs, all variants in the *APOE* region (chromosome 19, coordinates 44912079 to 45912079) were removed, while the PRRs with *APOE* include the variants rs429358 and rs7412. In the present study, we created PRSs including variants based on the *p* value thresholds *p <* 5e^−8^ and *p <* 1e^−5^ (referred to as 5e^−8^ PRS 1e^−5^ PRS). The third AD-PRS is based on 39 genetic variants (41 variants when including *APOE*) and was validated for the first time in a study by de Rojas et al. [28]. Five of the variants included in the original version of the PRS were missing in the ADNI data, leaving 34 and 36 SNPs respectively. The non-*APOE* version of this PRS includes six novel, three confirmed, and 30 previously reported genome-wide significant findings. All PRSs were calculated as the sum of the β-coefficient multiplied with the number of effect alleles of each genetic variant and then standardized. Genetic variants included in the different PRSs are shown in Additional file 1. Median INFO score of the variants used in the 5e^−8^ PRS and the 1e^−5^ PRSs was 0.99 (range 0.69–1.00), and for the variants in the 34-SNP PRS it was 0.99 (range 0.59–1.00). Prior to the PRS calculation 362 individuals who were part of IGAP were removed to avoid overfitting due to non-independency of the GWAS discovery data and the target data, generating a final sample of 962 individuals. We also included the 31-SNP+*APOE* polygenic hazard score (PHS), originally developed by Desikan et al. [29], that is available in the ADNI database. To facilitate comparison of results based on the PRSs, the PHS was standardized.

### Statistical analyses

Values on plasma p-tau181 were available for 826 of the individuals with PRSs and 822 of those with the PHS. Prior to the statistical analyses, outliers with plasma p-tau181 values more than 3 standard deviations (SDs) above or below the average of the whole population with plasma data were excluded (*n* = 6 of those with genetic data), and remaining values were logarithmized to meet normality assumption. Further, two individuals with missing information on diagnostic status in the ADNI database were excluded.

Sample characteristics at the plasma collection baseline (defined as an individual’s first visit with plasma data), among individuals with genetic data, were compared using chi-square test (categorical variables) and ANOVA (continuous variables). Associations between AD-PRSs and diagnostic status at plasma collection baseline were investigated using logistic regression models adjusted for sex, age, and seven PCs to correct for population stratification. Associations between AD-PRSs and plasma p-tau181 baseline values in the total sample were investigated using linear regression models adjusted for sex, age, diagnostic status (CU, MCI, or AD dementia), and seven PCs. Associations between AD-PRSs and plasma p-tau181 in the different diagnostic groups were analyzed using linear regression models adjusted for sex, age, and seven PCs. Further, the analyses in the total sample were repeated after stratifying the sample into Aβ positives and negatives. A significance level of < 0.05 was applied, but the results were also validated in relation to a Bonferroni corrected significance level of < 0.0083 (based on analyses performed in six different groups (i.e., total sample, CU, MCI, AD dementia, Aβ positives and negatives). Post hoc power analyses using effect sizes based on the associations seen in this study were performed. The statistical analyses were done in SPSS 26 and SAS 9.4.

## Results

Characteristics of the sample used in this study are shown in Table 1. The diagnostic groups included differed significantly in age, sex, plasma p-tau181 levels, and *APOE* ε4 and Aβ pathology status. Stratification based on Aβ pathology status showed similar results, except that age did not differ between diagnostic groups in Aβ positives. All versions of *APOE* PRSs, and the PHS, were associated with both AD and MCI status. Regarding the non-*APOE* PRSs, the 1e^−5^ PRS was associated with AD and MCI, the 34-SNP PRS was associated with AD, and the 5e^−8^ PRS was neither associated with AD nor MCI.

**Table 1:** Characteristics of the sample with genetic and plasma p-tau 181 data

	CU (***n*** = 236)	MCI (***n*** = 434)	AD (***n*** = 148)	
				*p*^a^
Age in years, mean (SD)	73.8 (6.1)	72.6 (7.8)	75.5 (7.9)	**1e−4**
Sex (women), *n* (%)	132 (55.9)	181 (41.7)	56 (37.8)	**3e−4**
*APOE ε4*, *n* (%)	69 (29.2)	176 (40.6)	101 (68.2)	**2e−12**
plasma p-tau 181, mean (SD)	14.9 (8.7)	18.3 (11.0)	23.6 (8.2)	**2e−15**
Years of education, mean (SD)	16.6 (2.7)	16.1 (2.8)	16.0 (2.7)	0.05
Aβ positivity^b^, *n* (%)	45 (21.0)	176 (45.6)	101 (77.1)	**2e−23**
*APOE* PRSs, median (min, max)				*p*^c^ *p*^d^
34-SNP PRS	− 0.63 (− 2.22, 2.04)	− 0.15 (− 1.74, 2.85)	0.45 (− 1.94, 2.89)	–
OR (95% CI)	ref	1.66 (1.37–2.02)	2.97 (2.24–3.95)	**3e−7** **5e−14**
5e−8 PRS	− 0.62 (− 2.48, 1.91)	− 0.20 (− 1.90, 2.52)	0.41 (− 1.83, 2.64)	–
OR (95% CI)	ref	1.69 (1.39–2.05)	2.88 (2.18–3.80)	**2e−7** **1e−13**
1e−5 PRS	− 0.56 (− 2.35, 2.08)	− 0.17 (− 2.26, 2.98)	0.40 (− 2.08, 3.14)	–
OR (95% CI)	ref	1.73 (1.42–2.11)	3.20 (2.37–4.31)	**4e−8** **2e−14**
PHS	− 0.66 (− 2.39, 2.03)	− 0.23 (− 1.89, 2.45)	0.48 (− 1.96, 2.78)	–
OR (95% CI)	ref	1.76 (1.44–2.15)	3.31 (2.46–4.45)	**3e−8** **2e−15**
non-*APOE* PRSs, median (min, max)				*p*^c^ *p*^d^
34-SNP PRS	− 0.14 (− 2.49, 2.88)	− 0.02 (− 2.32, 2.75)	− 0.03 (− 2.69, 3.62)	–
OR (95% CI)	ref	1.11 (0.94–1.30)	1.26 (1.02–1.56)	0.2 **0.04**
5e−8 PRS	− 0.12 (− 2.36, 2.92)	− 0.02 (− 3.33, 3.06)	− 0.01 (− 2.47, 3.12)	–
OR (95% CI)	ref	1.13 (0.96–1.33)	1.21 (0.97–1.51)	0.2 0.1
1e−5 PRS	− 0.21 (− 2.58, 2.61)	− 0.02 (− 3.43, 2.90)	0.08 (− 2.53, 2.82)	–
OR (95% CI)	ref	1.21 (1.02–1.42)	1.34 (1.07–1.68)	**0.03** **0.01**
**Aβ pos (*n*** **= 322)**				*p*^a^
Age in years, mean (SD)	72.2 (5.3)	73.9 (6.8)	74.0 (7.9)	0.5
Sex (women), *n* (%)	32 (71.1)	70 (39.8)	45 (44.6)	**1e−3**
*APOE ε4*, *n* (%)	25 (55.6)	117 (66.5)	81 (80.2)	**6e−3**
Plasma p-tau 181, mean (SD)	18.5 (7.8)	23.0 (10.8)	25.0 (8.4)	**1e−3**
Years of education, mean (SD)	16.2 (2.5)	15.9 (2.8)	15.9 (2.7)	0.8
**Aβ neg (*n*** **= 409)**				*p*^a^
Age in years, mean (SD)	73.3 (6.3)	71.1 (8.0)	78.1 (6.2)	**9e−7**
Sex (women), *n* (%)	85 (50.3)	91 (43.3)	6 (20.0)	**8e−3**
*APOE ε4*, *n* (%)	37 (21.9)	74 (35.2)	9 (30.0)	**0.02**
Plasma p-tau 181, mean (SD)	14.3 (8.9)	14.5 (9.6)	19.2 (7.1)	**0.02**
Years of education, mean (SD)	16.7 (2.7)	16.4 (2.7)	16.2 (2.6)	0.4

Significant *p* values are shown in bold

^a^Based on ANOVA for continuous variables and chi-square test for categorical variables

^b^Aβ pathology status was missing for 87 individuals

^c^Based on logistic regression including cognitively unimpaired (ref) and MCI

^d^Based on logistic regression including cognitively unimpaired (ref) and AD

Associations were found between all versions of *APOE* PRSs, the PHS, and plasma p-tau181 levels in the total sample (Table 2). Associations, but weaker, were also seen with the non-*APOE* PRSs, and with the PHS adjusted for *APOE* ε4 status (Table 2). When the analyses were repeated after stratifying the sample according to diagnostic status, the *APOE* PRSs and the PHS were associated with plasma p-tau181 in all groups (CU, MCI, and AD dementia) (Table 2). The non-*APOE* PRS, and the PHS adjusted for *APOE* ε4 status, showed associations only in the MCI group. All associations, except for those with the non-*APOE* 1e^−5^ PRS and the one with the PHS adjusted for ε4 status in MCI, would remain after Bonferroni correction for multiple testing (*p* value limit < 0.0083). Comparing the different versions of PRSs, and the PHS, indicated a relatively similar level of performance, with slightly lower *p* values for the 34-SNP PRS and the 5e^−8^ PRS.

**Table 2:** AD-PRSs versus log plasma p-tau181 in the total sample and by diagnostic status

	***APOE*** PRS				Non-***APOE*** PRS			
	Total sample (*n* = 818)							
**PRS version**	*β*	SE	95% CI	*p*	*β*	SE	95% CI	*p*
34-SNP PRS	0.19	0.02	0.14–0.23	**2e−17**	0.06	0.02	0.02–0.10	**2e−3**
5e−8 PRS	0.19	0.02	0.15–0.23	**3e−18**	0.08	0.02	0.03–0.12	**3e−4**
1e−5 PRS	0.18	0.02	0.13–0.22	**8e−16**	0.05	0.02	0.004–0.09	**0.03**
PHS*	0.18	0.02	0.13–0.22	**7e−15**	0.11	0.04	0.03–0.18	**8e−3**
	Cogn unimpaired (*n* = 236)							
	*β*	SE	95% CI	*p*	*β*	SE	95% CI	*p*
34-SNP PRS	0.16	0.05	0.05–0.27	**3e−3**	0.02	0.04	− 0.07 to 0.10	0.7
5e−8 PRS	0.20	0.05	0.09–0.31	**3e−4**	0.08	0.04	− 0.003 to 0.17	0.06
1e−5 PRS	0.18	0.06	0.07–0.28	**2e−3**	0.04	0.05	− 0.05 to 0.13	0.4
PHS*	0.19	0.06	0.08–0.30	**1e−3**	0.13	0.10	− 0.08 to 0.33	0.2
	MCI (*n* = 434)							
	*β*	SE	95% CI	*p*	*β*	SE	95% CI	*p*
34-SNP PRS	0.21	0.03	0.15–0.27	**8e−13**	0.09	0.03	0.04–0.15	**1e−3**
5e−8 PRS	0.21	0.03	0.15–0.26	**2e−12**	0.08	0.03	0.03–0.14	**1e−4**
1e−5 PRS	0.19	0.03	0.14–0.25	**4e−11**	0.06	0.03	0.004–0.12	**0.04**
PHS*	0.18	0.03	0.13–0.24	**2e−9**	0.11	0.06	0.004–0.22	**0.04**
	AD (*n* = 148)							
	*β*	SE	95% CI	*p*	*β*	SE	95% CI	*p*
34-SNP PRS	0.13	0.03	0.07–0.19	**6e−5**	0.03	0.03	− 0.03 to 0.09	0.4
5e−8 PRS	0.12	0.03	0.06–0.18	**7e−5**	0.03	0.03	− 0.04 to 0.09	0.4
1e−5 PRS	0.13	0.03	0.07–0.19	**7e−5**	0.03	0.03	− 0.04 to 0.09	0.4
PHS*	0.12	0.03	0.06–0.18	**1e−4**	0.06	0.05	− 0.04 to 0.15	0.2

Results within the total sample are adjusted for sex, age, principal components, and diagnostic status. Results within the diagnostic groups are adjusted for sex, age, and principal components. Significant *p* values are shown in bold

*Values presented in the “non-*APOE* PRS column” are adjusted for ε4-carriership instead of excluding *APOE*

Analyses of the total sample stratified into Aβ-positive and Aβ-negative individuals showed associations between the *APOE* PRSs, and the PHS, within both groups. The non-*APOE* PRSs (34-SNP PRS and 1e^−5^ PRS), and the PHS adjusted for *APOE* ε4 status, were associated with plasma p-tau181 levels among Aβ positives only (Table 3). The associations with the *APOE* scores, but not those with the non-*APOE* scores, would remain after Bonferroni correction for multiple testing (*p* value limit *p* < 0.0083). Once again, the different versions of PRSs, and the PHS, performed relatively similar, with slightly lower *p* values for the 34-SNP PRS and the 5e^−8^ PRS. Further, stratification of Aβ-positive and Aβ-negative individuals based on diagnostic status showed associations between the *APOE* PRSs, the PHS, and plasma p-tau181 in both Aβ positives and negatives in all diagnostic groups (Aβ positives: *p* = 5e^−3^–0.02, Aβ negatives: *p* = 1e^−3^–0.04) except for the AD group among Aβ positives. The non-*APOE* scores were borderline associated (*p* = 0.05–0.07) only in Aβ positives with MCI.

**Table 3:** AD-PRSs versus log plasma p-tau181 in Aβ-positive and Aβ-negative individuals

	***APOE*** PRS				Non-***APOE*** PRS			
	Aβ pos (*n* = 322)							
**PRS version**	*β*	SE	95% CI	*p*	*β*	SE	95% CI	*p*
34-SNP PRS	0.10	0.03	0.05–0.15	**7e**−**5**	0.06	0.02	0.01–0.10	**0.02**
5e−8 PRS	0.10	0.03	0.05–0.15	**9e**−**5**	0.05	0.02	0.01–0.10	**0.02**
1e−5 PRS	0.10	0.03	0.05–0.15	**2e**−**4**	0.05	0.02	− 0.001 to 0.09	0.06
PHS*	0.10	0.03	0.05–0.22	**2e**−**4**	0.09	0.04	0.01–0.17	**0.02**
	Aβ neg (*n* = 409)							
	*β*	SE	95% CI	*p*	*β*	SE	95% CI	*p*
34-SNP PRS	0.15	0.04	0.07–0.23	**2e**−**4**	0.01	0.03	− 0.05 to 0.08	0.7
5e−8 PRS	0.17	0.04	0.09–0.25	**5e**−**5**	0.04	0.04	− 0.04 to 0.10	0.3
1e−5 PRS	0.14	0.04	0.06–0.22	**1e**−**3**	−0.003	0.03	− 0.07 to 0.06	0.9
PHS*	0.14	0.04	0.06–0.15	**1e**−**3**	0.08	0.08	− 0.07 to 0.24	0.3

Results are adjusted for sex, age, principal components, and diagnostic status. Significant *p* values are shown in bold

*Values presented in the “non-*APOE* PRS column” are adjusted for ε4-carriership instead of excluding *APOE*

Post hoc power analyses based on effect sizes seen for the associations in this study showed that we had a power between 90 and > 99% to detect these effects in the total sample, CU group, and MCI group, while we had a power of 70% in the AD group. In the Aβ pathology stratified analyses, we had a power between 75 and > 99%.

To investigate if the associations seen between the non-*APOE* PRSs and plasma ptau-181 were beyond (independent of) *APOE*, we repeated the analyses of the non-*APOE* PRS vs plasma p-tau181 after adjusting for *APOE* ε4 status within the regression model. The significant *p* values within the total sample and the group of Aβ positives remained at exactly the same level, while significant *p* values within the MCI-group increased slightly (non-*APOE* 34-SNP PRS: *p* = 3e^−3^, non-*APOE* 5e^−8^ PRS: *p* = 8e^−3^). To study this in even more detail, we performed stratified analyses, based on *APOE* ε4 carrier status, within the total sample. We found that the non-*APOE* 34-SNP PRS and the non-*APOE* 5e^−8^ PRS were significantly associated with plasma p-tau181 in both ε4-carriers (*p* = 0.02–0.04) and non-carriers (*p* = 7e^−3^–0.03).

## Discussion

In this study, we investigated the relation between AD-PRSs and plasma p-tau181. Associations with both *APOE* PRSs and non-*APOE* PRSs were seen in the total sample including both symptomatic (i.e., MCI and AD dementia) and cognitively unimpaired individuals. The associations with the non-*APOE* PRSs could be seen in both carriers and non-carriers of the *APOE* ε4 allele. In stratified analyses, the *APOE* PRSs were associated with plasma p-tau181 in all diagnostic groups (CU, MCI, and AD dementia), while the non-*APOE* PRSs were associated with plasma p-tau181 only in the MCI group. The *APOE* PRSs showed similar results in Aβ- positive and Aβ-negative individuals, while the non-*APOE* PRSs were associated with plasma p-tau181 in Aβ positives only. All results were similar when using a pre-calculated PHS available in the ADNI database.

So far, studies of the relation between AD-PRSs and plasma p-tau181 are lacking. Previous CSF-based results of most relevance for comparison with our plasma-based results are those presented in a recent study of polygenic burden on AD pathology in ADNI [16]. Similar to our finding, the authors reported that CSF p-tau181 was independently (after correction for *APOE*) associated with the 31-SNP polygenic hazard score (PHS) (including *APOE* effects) used in our study. Neither results based on stratification by diagnostic status, nor by Aβ pathology status, were presented.

In contrast to our findings, results in a paper on the longitudinal Australian Imaging, Biomarkers and Lifestyle (AIBL) Study of Ageing showed no association between a 21-SNP PRS and CSF p-tau levels in a small (*n* = 58) combined sample of cognitively normal and AD individuals [30]. This was the case both with and without inclusion of *APOE* in the PRS. When only considering cognitively normal individuals (*n* = 42), an association was seen with the non-*APOE* PRS. Another study reported no associations between a 19-SNP PRS (both with and without inclusion of *APOE*) and CSF p-tau levels in a sample of cognitively normal individuals enriched for a parental history of AD [31].

Other studies in relation to CSF are performed within MCI or AD only. A study on individuals with MCI showed a similar result as ours (i.e., an association with p-tau) when analyzing an 18-SNP non-*APOE* PRS in 391 individuals from two European cohorts [32]. In AD patients (*n* = 338), a study by Sleegers et al. [33] reported a correlation between a non-*APOE* 21-SNP PRS and CSF p-tau levels, while no association was seen when *APOE* was included in the score. In contrast, another study on AD patients (*n* = 890) [34], performing analyses with a relatively similar PRSs (including and excluding *APOE*), reported no associations with CSF p-tau. Both studies in AD contradict our results in the AD sub-group in ADNI (i.e., association with the *APOE* PRS and no association with the non-*APOE* PRS). In summary, studies of the relation between AD-PRSs and p-tau181 levels, including our novel plasma data, show mixed results, especially in cognitively normal and AD individuals. Possible explanations are differences in diagnostic/disease stage, and sometimes also differences in genetic variants included in the PRSs. Importantly, our results in plasma replicate a previous report in CSF within the same cohort [16]. Moreover, we included three versions of PRSs and they performed relatively similar. We also included the PHS, which was the polygenic score that performed best in relation to CSF biomarkers in the ADNI-study by Altmann et al. [16].

Our findings suggest that genetic risk for AD, driven by *APOE*, is associated with plasma p-tau181 in all diagnostic groups, while genetic risk for AD beyond *APOE* is associated with plasma p-tau181 only in the MCI group. Results from Karikari et al. [3] indicate that plasma p-tau181 increases during early stages of tau pathology accumulation, but reaches a plateau in cases with moderate to severe pathology. In agreement, the performance of plasma p-tau to identify AD pathology is very high in later disease stages [7]. In view of these results, one could hypothesize that genetic risk factors for AD, especially those with smaller effect than *APOE*, are of greater importance in relation to p-tau181 during the time when the level of the biomarker changes the most.

Considering the influence of Aβ pathology on the relation between AD-PRS and plasma p-tau181, the PRSs (and PHS) including *APOE* seem to be associated with plasma p-tau181 independent of Aβ pathology status, while the non-*APOE* scores were associated only in Aβ-positive individuals. One possible explanation may be that the relatively weak effect of the non-*APOE* PRSs in AD makes it easier to identify an association between the non-*APOE* PRSs and plasma p-tau181 in Aβ positives, which constitute a genuine AD group harboring brain amyloidosis. In contrast, the very strong effect of *APOE* in AD may allow for identifying an association between the *APOE* PRSs and plasma p-tau181 levels also in a mixed population of cases with and without brain amyloidosis. Stratifying Aβ-positive and Aβ-negative individuals into diagnostic groups showed that the associations between the *APOE* PRSs and plasma p-tau181 were relatively independent of diagnostic status. The associations between the non-*APOE* PRSs and plasma p-tau181 within Aβ positives could, however, only be seen (at borderline significance level) in individuals with MCI. This indicates that having MCI and being Aβ-positive is the most vulnerable combination in relation to the association between genetic risk for AD beyond *APOE* and plasma p-tau181. Previous studies have shown that p-tau181 increases already in response to Aβ pathology and then further in a stepwise manner with more advanced Braak stage [3–5, 7]. P-tau181 signaling already in response to Aβ positivity is in line with our results for the non-*APOE* PRSs (i.e., association with ptau181 in MCI and Aβ positive individuals).

Overall, our results support the importance of using biomarkers to identify Aβ and tau pathology in studies assessing genetic risk for AD. This study shows that we can learn more about the pathophysiology and disease processes in AD by combining information from well-validated fluid biomarkers and PRSs. High-risk individuals on the basis of genetics who do not increase in p-tau as expected may be protected in some way; finding such resilience factors is important (and vice versa: low risk individuals who get p-tau increase could have lifestyle-related risk factors). This will be important future studies, likely requiring larger cohorts. Further, utilizing information based on the combination of PRSs and blood biomarkers may be a useful way to detect and enrich clinical trials with individuals at higher risk to develop symptomatic AD.

### Limitations

This study has some limitations that have to be addressed. The sample size is relatively small, making it difficult to find associations that survive correction for multiple testing in some of the stratified analyses. The majority of the associations seen in this study would remain after Bonferroni correction, but the associations with the non-*APOE* scores in Aβ positives would not and should therefore be interpreted with caution. Due to the lack of previous studies (i.e., previous effect sizes) of the relation between AD-PRSs and plasma p-tau181, a priori power analyses were not possible to do. However, post hoc power analyses based on effect sizes for the associations in our study showed that we had a relatively high power to detect these effect levels (using a *p* value limit of < 0.05), both in the total sample and after stratifying on either diagnostic or Aβ pathology status. Still, stratifying on both diagnostic status and Aβ pathology status generated small groups and these results have to be interpreted with caution. Further, the sample size of the different diagnostic groups differs. However, inspection of the β-values and standard errors indicates that associations seen in only one of the diagnostic groups (i.e., associations between the non-*APOE* PRSs, the PHS, and MCI) are not merely a result of a larger sample size. Moreover, due to the very limited number of cohorts with both GWAS and plasma p-tau181 data, no replication sample of comparable size could be feasibly included in this study. Finally, the ADNI cohort could not be considered a representative population-based sample, which limits the possibility of generalizing the results to the broader population.

## Conclusions

Polygenic risk for AD including *APOE* was found to associate with plasma p-tau181 independent of diagnostic status and Aβ pathology status, while polygenic risk for AD beyond *APOE* was associated with plasma p-tau181 only in MCI patients and Aβ-positive individuals. These results extend the knowledge about the relation between genetic risk for AD and p-tau181 and further support the usefulness of plasma p-tau181 as a biomarker of AD.

## Supplementary Information

**Additional file 1.**

## Data Availability

The data used in this article can be found in the ADNI database (http://adni.loni.usc.edu) [17].

## Abbreviations

AD: Alzheimer’s disease
ADNI: Alzheimer’s Disease Neuroimaging Initiative
Aβ: Amyloid-β
CSF: Cerebrospinal fluid
CU: Cognitively unimpaired
GWAS: Genome-wide association study
IBD: Identity-by-decent
LD: Linkage disequilibrium
MCI: Mild cognitive impairment
MAF: Minor allele frequency
p-tau181: Phosphorylated tau181
PHS: Polygenic hazard score
PRS: Polygenic risk score
PET: Positron emission tomography
PCs: Principal components
Simoa: Single molecule array
SNPSs: Single nucleotide polymorphisms
SUVR: Standardized uptake value ratios
