# Supplementary material for: Association between polygenic risk score of Alzheimer’s disease and plasma phosphorylated tau in individuals from the Alzheimer’s Disease Neuroimaging Initiative
Source: Alzheimers Res Ther. 2021 Jan 8;13:17. doi: 10.1186/s13195-020-00754-8 (PMC7792087; doi:10.1186/s13195-020-00754-8)
Supplement: Supplementary file 1 — Additional file 1. [file 13195_2020_754_MOESM1_ESM.docx]

**Additional table 1.** Genetic variants included in the AD-PRSs.

| **34-SNP PRS^a^** |  |  |  | **1e-5 PRS^b^** |  |  |  |
| --- | --- | --- | --- | --- | --- | --- | --- |
| SNP | Closest gene | Chrom | Pos | SNP | Closest gene | Chrom | Pos |
| rs4844610 | *CR1* | 1 | 207802552 | rs12562818 | *KIF21B* | 1 | 200952336 |
| rs6733839 | *BIN1* | 2 | 127892810 | rs3134617 | *MYCL* | 1 | 40361843 |
| rs10933431 | *INPP5D* | 2 | 233981912 | rs4844610* | *CR1* | 1 | 207802552 |
| rs4351014 | *HS3ST1* | 4 | 11027619 | rs10933431* | *INPP5D* | 2 | 233981912 |
| rs143332484 | *TREM2* | 6 | 41129207 | rs35695568 | *FSIP2* | 2 | 186794162 |
| rs75932628 | *TREM2* | 6 | 41129252 | rs6733839* | *BIN1* | 2 | 127892810 |
| rs9381040 | *TREML2* | 6 | 41154650 | rs4574296 | *CADM2* | 3 | 86347434 |
| rs9381564 | *CD2AP* | 6 | 47443806 | rs7617515 | *ZNF852* | 3 | 44539183 |
| rs1859788 | *PILRA* | 7 | 99971834 | rs9880254 | *CLEC3B* | 3 | 45098958 |
| rs56402156 | *EPHA1* | 7 | 143103481 | rs2725479 | *GASK1B* | 4 | 158827027 |
| rs73223431 | *PTK2B* | 8 | 27219987 | rs190982 | *MEF2C-AS1* | 5 | 88223420 |
| rs9331896 | *CLU* | 8 | 27467686 | rs31930 | *OTULIN* | 5 | 14678996 |
| rs34674752 | *SHARPIN* | 8 | 145154222 | rs4150233 | *HBEGF* | 5 | 139714837 |
| rs34173062 | *SHARPIN* | 8 | 145158607 | rs77790943 | *ADCY10P1* | 6 | 41070941 |
| rs7920721 | *ECHDC3* | 10 | 11720308 | rs9389138 | *SCLA12* | 6 | 134389525 |
| rs3740688 | *SPI1* | 11 | 47380340 | rs9473117* | *CD2AP* | 6 | 47431284 |
| rs1582763 | *MS4A2* | 11 | 60021948 | rs10808026* | *EPHA1* | 7 | 143099133 |
| rs3851179 | *PICALM* | 11 | 85868640 | rs12539172* | *NYAP1* | 7 | 100091795 |
| rs11218343 | *SORL1* | 11 | 121435587 | rs34213597 | *AZGP1* | 7 | 99591021 |
| rs17125924 | *FERMT2* | 14 | 53391680 | rs4723711 | *NME8* | 7 | 37844263 |
| rs11623019 | *SLC2A4* | 14 | 92936971 | rs231144 | *TRPS1* | 8 | 116416131 |
| rs593742 | *ADAM10* | 15 | 59045774 | rs4735340 | *NDUFAF6* | 8 | 95976251 |
| rs117618017 | *APH1B* | 15 | 63569902 | rs73223431* | *PTK2B* | 8 | 27219987 |
| rs7185636 | *IQCK* | 16 | 19808163 | rs9331896* | *CLU* | 8 | 27467686 |
| rs4985556 | *IL34* | 16 | 70694000 | rs10811519 | *IFNA22P* | 9 | 21266497 |
| rs12444183 | *PLCG2* | 16 | 81773209 | rs6559689 | *RASEF* | 9 | 85450616 |
| rs75511804 | *SCIMP* | 17 | 5138304 | rs142366127 | *REEP3* | 10 | 66004362 |
| rs616338 | *ABI3* | 17 | 47297297 | rs4934028 | *MAT1A* | 10 | 82038779 |
| rs4311 | *ACE* | 17 | 61560763 | rs71501966 | *SH2D4B* | 10 | 82381685 |
| rs72835061 | *CHRNE* | 17 | 4805437 | rs7920721* | *ECHDC3* | 10 | 11720308 |
| rs3752231 | *ABCA7* | 19 | 1043638 | rs10751667 | *AP2A2* | 11 | 941941 |
| rs12459419 | *CD33* | 19 | 51728477 | rs11038990 | *CKAP5* | 11 | 46804761 |
| rs7412 | *APOE* | 19 | 45412079 | rs34467936* | *NUP160* | 11 | 47915299 |
| rs429358 | *APOE* | 19 | 45411941 | rs3740688* | *SPI1* | 11 | 47380340 |
| rs6024870 | *CASS4* | 20 | 54997568 | rs3781832 | *SORL1* | 11 | 121436270 |
| rs2154481 | *APP* | 21 | 27473875 | rs3851179* | *PICALM* | 11 | 85868640 |
|  |  |  |  | rs7933202* | *MS4A6A* | 11 | 59936926 |
|  |  |  |  | rs7295246 | *ADAMTS20* | 12 | 43967677 |
|  |  |  |  | rs12881735* | *SLC24A4* | 14 | 92932828 |
|  |  |  |  | rs17125924* | *FERMT2* | 14 | 53391680 |
|  |  |  |  | rs10467994 | *SPPL2A* | 15 | 51008687 |
|  |  |  |  | rs593742 | *ADAM10* | 15 | 59045774 |
|  |  |  |  | rs11538963 | *MTSS2* | 16 | 70696272 |
|  |  |  |  | rs62039712 | *MAF* | 16 | 79355857 |
|  |  |  |  | rs7185636 | *IQCK* | 16 | 19808163 |
|  |  |  |  | rs2632516 | *TSPOAP1-AS1* | 17 | 56409089 |
|  |  |  |  | rs79013565 | *PITPNA* | 17 | 1464466 |
|  |  |  |  | rs12459419 | *CD33* | 19 | 51728477 |
|  |  |  |  | rs1560118 | *ELL* | 19 | 18570035 |
|  |  |  |  | rs17878252 | *FBXO46* | 19 | 46234155 |
|  |  |  |  | rs3752246* | *ABCA7* | 19 | 1056492 |
|  |  |  |  | rs429358* | *APOE* | 19 | 45411941 |
|  |  |  |  | rs7412* | *APOE* | 19 | 45412079 |
|  |  |  |  | rs75364577* | *CNN2* | 19 | 1028149 |
|  |  |  |  | rs6024870* | *CASS4* | 20 | 54997568 |
|  |  |  |  | rs2830500 | *ADAMTS1* | 21 | 28156856 |
|  |  |  |  | rs909441 | *LOC100506403* | 21 | 37069610 |

^a^Based on the PRS validated in de Rochas et al. 2019.

^b^Based on summary statistics from Kunkle et al. 2019.

*Included in the 5e-8 PRS.
